# A rare intronic c.2654+1G>A mutation in CSF1R-microglial encephalopathy: a case report

**DOI:** 10.3389/fgene.2025.1593964

**Published:** 2025-08-15

**Authors:** HongYan Wu, JingYu Shi, XiaoShan Wang, Mei Yang, Jing Cai

**Affiliations:** ^1^ Department of Neurology, Guizhou University of Traditional Chinese Medicine, Guiyang, China; ^2^ Department of Neurology, First Affiliated Hospital of Guizhou University of Traditional Chinese Medicine, Guiyang, China

**Keywords:** CSF1R (colony stimulating factor 1 receptor), microglia, dementia, gene, magnetic resonance imaging

## Abstract

**Objective:**

We report a case of CSF1R-microglial encephalopathy associated with a rare intronic c.2654 + 1G>A mutation, featuring negative diffusion-weighted imaging (DWI) findings and a cerebrospinal fluid (CSF) biomarker profile indicative of Alzheimer’s disease-related changes, and we explore the associations between genetic mutations, CSF biomarker alterations, and neuroimaging manifestations.

**Methods:**

This study documents the demographic data, detailed medical history, and clinical manifestations of a patient with CSF1R-microglial encephalopathy. The medical histories of some family members were collected, and the proband underwent whole-exome sequencing (WES) for diagnostic confirmation.

**Results:**

The patient, a 53-year-old woman, presented with early-onset cognitive decline, personality changes, and behavioral abnormalities. Neuropsychological testing revealed severe cognitive impairment, and the CSF biomarker profile suggested Alzheimer’s disease-related changes. Cranial MRI showed bilateral, symmetric deep white matter changes, brain atrophy (including corpus callosum thinning), and low signal intensity on DWI. Family history revealed that 3 out of 19 individuals across four generations, including the proband, her aunt, and her sister, developed dementia and progressed to severe cognitive impairment rapidly. WES analysis revealed a heterozygous c.2654 + 1G>A variant in the *CSF1R* gene (NM_005211.3), confirming a diagnosis of CSF1R-microglial encephalopathy caused by a dominant autosomal mutation in exon 20 of the *CSF1R* gene.

**Conclusion:**

CSF1R-microglial encephalopathy is a progressive disorder with diverse early clinical presentations, making it prone to misdiagnosis and delayed treatment. This case suggests that, contrary to previous findings, negative DWI results should not exclude CSF1R-microglial encephalopathy. In addition, CSF biomarker profiles in patients with CSF1R-microglial encephalopathy may exhibit Alzheimer’s disease-related changes. Early genetic testing is critical, and for genetically linked diseases, testing other family members can help ensure early diagnosis and intervention.

## 1 Introduction

Mutations in the colony-stimulating factor 1 receptor (*CSF1R*) gene can lead to adult-onset primary central nervous system microglial disease, which disrupts axon-glia integrity and results in progressive and ultimately fatal white matter encephalopathy (Rademakers et al., 2011), accounting for approximately 10% of adult-onset idiopathic leukodystrophy, making it one of the most common causes of this condition (Lynch et al., 2016a). *CSF1R* gene mutations have been reported to cause hereditary diffuse leukoencephalopathy with spheroids (HDLS) and pigmentary orthochromatic leukodystrophy (POLD), both of which involve genetically-driven diffuse white matter lesions. Due to their similar pathological features, these conditions are collectively referred to as adult-onset leukodystrophy with neuroaxonal spheroids and pigmented glia (ALSP) (Wider et al., 2009). The known pathogenic genes for ALSP include not only the autosomal dominant *CSF1R*, but also the autosomal recessive *AARS2* (alanyltRNA synthetase 2) (Lynch et al., 2016b). ALSP can develop at any age, with no significant difference in incidence between males and females; however, the mean age at onset is earlier in females than in males (Konno et al., 2017). To date, more than 100 mutations in the *CSF1R* gene have been reported in patients with ALSP (Chitu et al., 2022). The *CSF1R* gene is located on chromosome 5q32 and consists of 22 exons that encode membrane-associated proteins. *CSF1R* functions as a receptor tyrosine kinase and is structurally composed of five immunoglobulin-like domains in its extracellular region, a transmembrane domain (TM), a juxtamembrane domain (JMD), a kinase insert domain (KID), and two tyrosine kinase domains (TKDs). The majority of pathogenic mutations have been identified within the tyrosine kinase domains encoded by exons 12–21, while no disease-related mutations have yet been reported in exon 16 (Wang et al., 2021). The c.2654 + 1G>A splice site mutation is a rare but pathogenic variant among known splicing defects. Furthermore, researchers have described the hereditary white matter disease caused by *CSF1R* gene mutations as CSF1R-microglial encephalopathy, which includes genetic, pathological, and clinical features to characterize and unify the diverse phenotypic spectrum of *CSF1R* mutations (Jiang et al., 2022). The initial symptoms of CSF1R-microglial encephalopathy often present as progressive cognitive decline, depression, apathy, anxiety, irritability, behavioral abnormalities, and personality changes, followed by or occurring concurrently with motor symptoms, such as muscle rigidity, bradykinesia, gait disturbances, and ataxia (Konno et al., 2018a; Rosenstein et al., 2022). The clinical manifestations closely resemble those of various neurological disorders, including multiple sclerosis, frontotemporal dementia, Parkinson’s disease, and atypical Parkinsonism. Patients with CSF1R-associated microglial encephalopathy typically exhibit characteristic high signal intensity in white matter on DWI sequences (Mickeviciute et al., 2022). However, there have been almost no reports of cases without significant white matter abnormalities on DWI. CSF1R-microglial encephalopathy presents with complex clinical features, and some patients exhibit atypical symptoms, making misdiagnosis common in clinical practice. Genetic testing is the preferred method for diagnosing CSF1R-microglial encephalopathy. Here, we report a case of *CSF1R* gene mutation with Alzheimer’s disease pathological changes and negative DWI. The patient carries a heterozygous splice site mutation in the CSF1R gene (c.2654 + 1G>A, NM_005211.3), accompanied by progressive cognitive decline and a CSF biomarker profile suggesting Alzheimer’s disease-related changes, with no white matter abnormalities detected on the DWI sequence.

## 2 Case report

### 2.1 Clinical data

**FIGURE 1:**
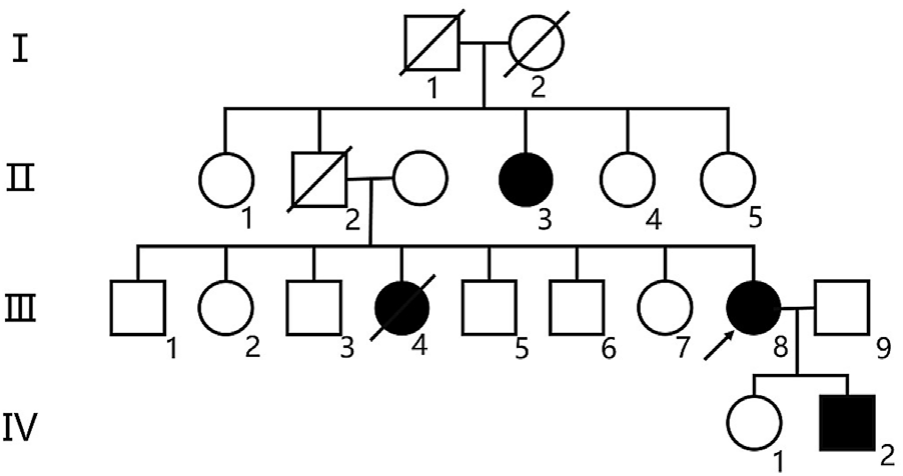
Pedigree chart. The Roman numerals on the left indicate the generation. Circles represent women, while squares represent men. The arrow points to the proband (patient III-8). Affected patients are shown by black symbols, while white symbols represent unaffected or at-risk individuals with an unknown phenotype.

The patient is a 53-year-old female who was admitted to the hospital on 4 July 2024, due to a 5-month history of memory decline, which worsened over the past week. According to the family members, the patient had no obvious triggers before the memory decline, which was mainly related to recent memory issues, such as forgetting the location of items, frequently calling family members and asking the same questions, as well as a decline in comprehension and executive abilities. This has affected daily life (e.g., forgetting to cook, a decrease in calculation ability, and making errors when shopping or paying for transportation). The patient also displayed personality changes (from being assertive and shrewd to becoming weak and submissive), changes in eating habits (increased craving for fatty meat since the onset of the disease), and compulsive personal hygiene management (washing clothes multiple times a day). The patient had not paid attention to or sought treatment for these symptoms, and there was no significant improvement. One week ago, the patient’s memory decline significantly worsened without an obvious trigger, with impaired time orientation, forgetfulness regarding the date, sluggish responses, low mood, and loss of interest in surrounding events. On 27 June 2024, the patient visited the outpatient department of this hospital, where she was prescribed Piracetam 0.4 g three times a day orally, but her condition did not improve significantly. To further complete relevant examinations and clarify the diagnosis, the patient was admitted on 4 July 2024. Since the onset of the disease, the patient’s eating and sleeping patterns have been poor. She can control spontaneous urination and defecation, and has experienced a weight loss of approximately 10 kg in the past year. The patient has no history of hypertension, diabetes, or coronary heart disease. There is no history of trauma or surgery. No history of exposure to chemicals, radiation, or toxic substances. No history of drug or food allergies, and there are no abnormalities in the patient’s personal, marital, reproductive, or menstrual history. The patient originates from a non-consanguineous Chinese family, spanning four generations, and consisting of 19 members. The family pedigree is presented in Figure 1. Three family members, including the proband, his aunt, and his sister, have all exhibited similar symptoms of cognitive impairment. The patient’s aunt (II-3) developed cognitive impairment at the age of 67 and was later diagnosed with Alzheimer’s disease at another hospital. She has now progressed to severe dementia, exhibiting symptoms such as not recognizing family members, not knowing her own identity, being unable to care for herself (requiring assistance for toileting, bathing, and eating), and being unable to control urination and defecation. The patient’s sister (III-4) developed cognitive impairment at the age of 35, with rapid progression of the disease. By 40, she had become unable to perform daily activities independently, eventually becoming bedridden. She experienced difficulty with dressing, eating, and was unable to control urination and defecation. She passed away at the age of 42. The 28-year-old son (IV-2) of the patient carries the c.2654 + 1G>A mutation in CSF1R, with normal cognitive function and no significant MRI abnormalities.

### 2.2 Neurological examination

The neurological results were as follows: clear-minded, without hallucinations, and clear speech. Higher cognitive functions are impaired, and the reaction times are slow. Recent memory decline, long-term memory preserved, and decreased executive function and logical reasoning abilities. Orientation and calculation abilities are notably diminished. There are difficulties in repetition, recognition, writing, and visual attention deficits. The patient cooperated with the examination. Cranial nerve findings were negative, and muscle strength and tone in the limbs were normal. The patient successfully completed the bilateral finger-nose test, the rapid alternating movement test, the heel-to-shin test, as well as the Romberg test. No tremors, seizures, or spasms were observed. Tendon reflexes were present in the limbs, with no pathological signs elicited. Meningeal irritation signs were negative.

### 2.3 Auxiliary examination

#### 2.3.1 Neuropsychological testing scales

The MMSE score is 5/30, with a memory/recall score of 0/0 and a significant decline in orientation, memory, calculation, language ability, and executive function. The MoCA score is 6/30, with obvious deficits in orientation, memory, calculation, comprehension, language, and executive function. The ADL score is 34 (suggesting mild impairment in activities of daily living), and the CDR score is 3 (Memory 3, Orientation 3, Judgment and Problem Solving 3, Community Affairs 3, Home and Hobbies 2, Personal Care 1); the NPI score is 16.

#### 2.3.2 Imaging examination

**FIGURE 2:**
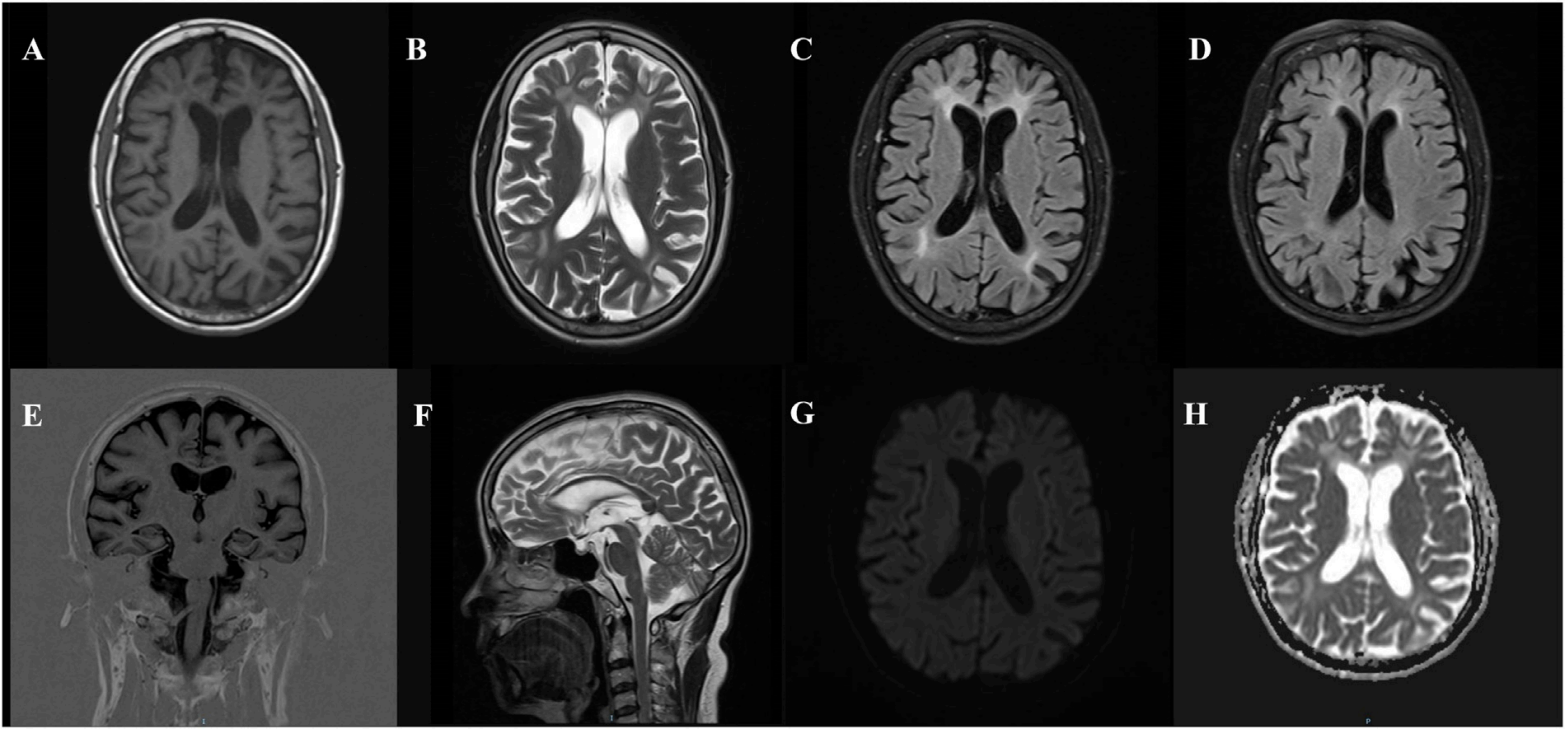
Brain MRI findings in a patient with *CSF1R* microglial encephalopathy. T1-weighted imaging **(A)**, T2-weighted imaging **(B)**, FLAIR **(C, D)** show symmetrical bilateral deep white matter changes, affecting the periventricular area, with varying degrees of brain atrophy (mainly in the posterior cortex), mild atrophy of the bilateral frontal and temporal lobes, T1 coronal **(E)** showing mild bilateral hippocampal atrophy, T2-weighted sagittal **(F)** showing thinning of the corpus callosum, DWI **(G)** showing low signal, and ADC **(H)** showing no obvious abnormalities.

Brain MRI indicates high signal intensity in the white matter (Fazekas score: grade 3). Cerebral atrophy with mild atrophy of both hippocampi. DWI reveals no abnormal diffusion restriction throughout the brain, and cranial SWI shows no significant abnormalities (Figure 2).

#### 2.3.3 Cerebrospinal fluid testing

Routine cerebrospinal fluid (CSF) tests and biochemical tests showed no significant abnormalities. Alzheimer’s disease CSF biomarkers: β-Amyloid protein 42, 88.56 pg/mL; β-Amyloid protein 42/β-amyloid protein 40, 0.082; Total Tau protein, 406.34 pg/mL (Table 1).

**TABLE 1:** Cerebrospinal fluid markers of AD.

Results of cerebrospinal fluid markers in Alzheimer’s disease			
Name		Result	Normal Reference value (pg/mL)
β-Amyloid protein 42	Aβ42	88.56↓	≥484
β-Amyloid protein 40	Aβ40	1,081.18	-
β-Amyloid protein 42/β-amyloid protein 40	Aβ42/Aβ40	0.082↓	≥0.099
Phosphorylated Tau protein	P-Tau(181p)	22.85	≤69.80
Total Tau protein	T-Tau	406.34↑	≤127.98
α-synuclein	α-Syn	2,226.72	≤5,167

#### 2.3.4 Genetic testing

**FIGURE 3:**
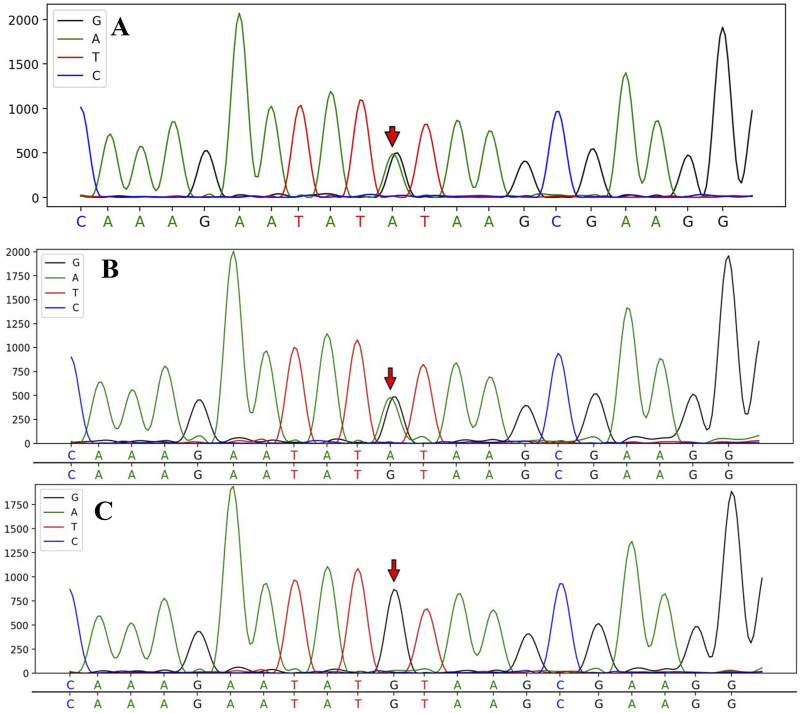
Sanger sequencing analysis of *CSF1R* gene mutations in the patient and her children. The sequencing analysis identified a splice mutation (c.2654 + 1G>A) in intron 20 of the *CSF1R* gene, which was present in the proband III-8 **(A)** and the proband’s son IV-2 **(B)**; however, no *CSF1R* gene mutation was detected in the proband’s daughter IV-1 **(C)**.

WES results identified a heterozygous c.2654 + 1G>A variant in the CSF1R gene (NM_005211.3), located in exon 20, in both the patient and their son. Whole exome sequencing covered over 95% of the target region, with an average sequencing depth of 220X, ensuring reliable variant detection across most regions. The percentage of target regions with an average depth greater than 1X was 99.72%, and those with a depth greater than 20X accounted for 99.61%. All analyses were performed using standard quality control and data filtering steps to ensure the accuracy and reliability of the variant detection. Specific quality control metrics include a total sequencing data of 14.5683 Gb and a Q30 value of 97.17%. (Figure 3).

#### 2.3.5 Laboratory examination

The results of the complete blood count, comprehensive biochemical panel, coagulation profile, immune panel, routine urine and stool tests, thyroid function tests, vasculitis markers, and infectious markers showed no significant abnormalities.

#### 2.3.6 Other examinations

The echocardiogram results indicate normal left ventricular wall motion at rest, mild tricuspid regurgitation, and normal left ventricular systolic and diastolic functions. No significant abnormalities were observed in the neck vascular ultrasound. The electromyogram results show abnormal P300, while the electroencephalogram results reveal no significant irregularities.

### 2.4 Medical diagnosis

This 53-year-old female patient presented with an insidious onset and progressively worsening symptoms, including recent memory decline, while retaining some long-term episodic memory. Decreased ability to perform daily living activities was noted. Neurological examination revealed specific features of Gerstmann syndrome (including acalculia and agraphia) and Balint syndrome (manifesting as visual attention deficits, including simultanagnosia). The patient also exhibited cognitive impairment, personality changes, and behavioral abnormalities. Brain MRI showed symmetric deep white matter alterations bilaterally, involving the periventricular regions, with varying degrees of cerebral atrophy (primarily in the posterior cortex), mild atrophy of the bilateral frontal and temporal lobes, mild atrophy of the bilateral hippocampi, and thinning of the corpus callosum. Diffusion-weighted imaging (DWI) revealed no abnormal restricted diffusion across the brain. Cerebrospinal fluid biomarkers showed reduced Aβ42, a decreased Aβ42/Aβ40 ratio, and increased t-tau, suggesting Alzheimer’s disease-related changes. These findings indicated pathological changes consistent with AD. As a result, the patient was initially diagnosed with posterior cortical atrophy at an early stage. However, the patient presented with an early onset of symptoms, and based on neuropsychological test results, it was noted that the patient’s cognitive function declined significantly within a short period. Furthermore, considering the family history of dementia, genetic testing was subsequently carried out, which revealed a heterozygous c.2654 + 1G>A variant in the *CSF1R* gene.

**FIGURE 4:**
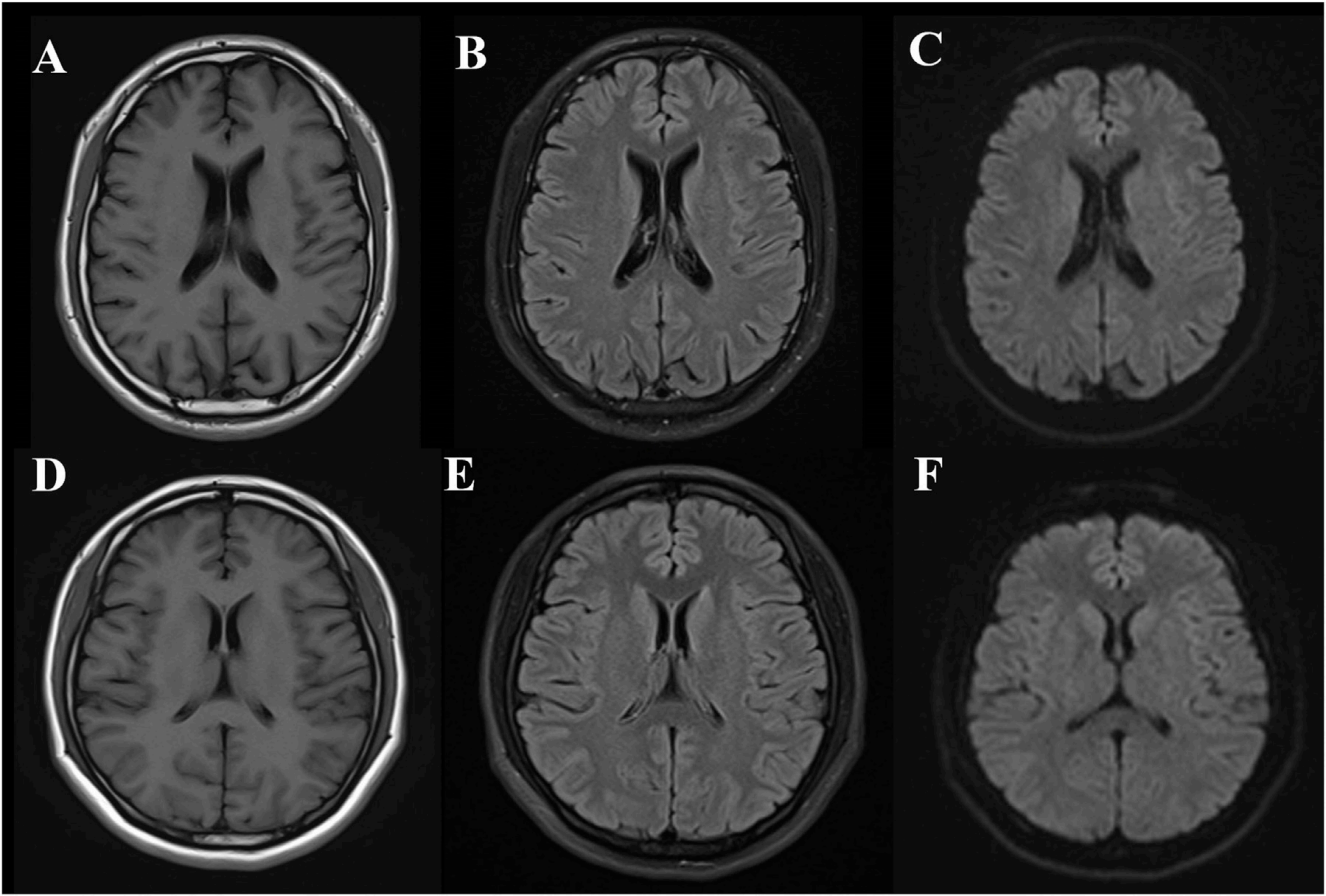
Brain MRI findings in the son and daughter of a CSF1R-microglial encephalopathy patient. The MRI of the patient’s son (IV-2) revealed no significant abnormalities in T1, T2 FLAIR **(A,B)** and DWI **(C) **while the MRI of the patient’s daughter (IV-1) revealed no significant abnormalities in T1, T2 FLAIR **(D,E)** and DWI **(F)**.

The patient’s son carries a heterozygous c.2654 + 1G>A mutation in the *CSF1R* gene, with no significant abnormalities observed on cranial MRI (Figure 4), normal EEG results, and self-reported normal cognitive function. His MMSE score is 27/30, and the MoCA score is 24/30, with a delayed recall score of 1/5, suggesting a deficit in delayed recall. The ADL score is 20 (indicating full independence in activities of daily living), and the CDR score is 0. The patient’s daughter did not carry a CSF1R gene mutation, and no significant abnormalities were observed on cranial MRI (Figure 4) or EEG. Her MMSE score was 29/30, MoCA score was 29/30, ADL score was 20 (indicating full independence in activities of daily living), and CDR score was 0.

In summary, the patient exhibits the core features of CSF1R-microglial encephalopathy: age of onset ≤60 years, cognitive impairment, autosomal dominant inheritance, bilateral symmetric deep white matter changes, brain atrophy (corpus callosum thinning), and a *CSF1R* gene mutation is present. Therefore, according to diagnostic criteria, a definitive diagnosis of CSF1R-microglial encephalopathy can be established (Konno et al., 2018b), and the patient was treated with memantine hydrochloride tablets (5 mg bid, titrated up to 10 mg bid), donepezil hydrochloride tablets (10 mg qn), and sodium oligomannate capsules (450 mg tid) as combination therapy. Three months after discharge, follow-up showed that the patient felt an improvement in their condition, with cognitive function being more stable and the frequency of repeated questions reduced. The MMSE score upon re-examination was 7/30, with a memory/recall score of 2/1. Memory has shown improvement compared to prior assessments, while orientation, calculation, language, and executive functions have remained stable. The MOCA score was 6/30, with notable deficits in orientation, memory, calculation, comprehension, language, and executive functions. The ADL score was 34 (suggesting mild impairment in activities of daily living), and the CDR score was 3 (Memory 3, Orientation 3, Judgment and Problem Solving 3, Community Affairs 3, Home and Hobbies 2, Personal Care 1); the NPI score was 16. Six months after discharge, the patient reported stable cognitive function, but demonstrated sluggish responses, poor concentration, and frequent interruptions while performing household tasks. Family members were required to remind the patient to continue with their tasks. The patient displayed a low mood and occasional irritability, often losing temper over trivial matters. The MMSE score upon re-examination was 11/30, with a memory/recall score of 3/3. Memory has shown further improvement compared to prior assessments, while orientation, calculation, language, and executive functions have remained stable. The MOCA score was 6/30, indicating significant deficits in orientation, memory, calculation, comprehension, language, and executive functions. The ADL score was 35 (suggesting mild impairment in activities of daily living), and the CDR score was 3 (Memory 3, Orientation 3, Judgment and Problem Solving 3, Community Affairs 3, Home and Hobbies 2, Personal Care 1); the NPI score was 17 (Table 2).

**TABLE 2:** Neuropsychological scale score.

Visit cycle*		V0 (2024.6.27)	V1 (2024.9.28)	V2 (2024.12.29)
Assessment Scale (score)	MMSE	5	7	11
	MoCA	6	6	6
	ADL	34	34	35
	CDR	3	3	3
	NPI	16	16	17

Note: *The guidelines recommend a visit cycle of once every 3 months.

## 3 Discussion

In this study, we present a case of CSF1R-microglial encephalopathy in a patient with a splice mutation (c.2654 + 1G>A) in the *CSF1R* gene, a family history of early-onset dementia, and a CSF biomarker profile suggesting Alzheimer’s disease-related changes. Despite these findings, the patient displayed only cognitive decline, with no evidence of abnormal white matter hyperintensities on DWI sequences. The patient’s age of onset was 53 years. While CSF1R-microglial encephalopathy typically presents between 40 and 50 years of age, studies indicate that the onset can range from 17 to 78 years (Wu et al., 2022). The patient initially presented with a decline in recent memory, followed by gradual deterioration in executive function, comprehension, orientation, and daily living abilities, accompanied by personality changes and behavioral abnormalities, which is consistent with the characteristics reported in previous cases of CSF1R-microglial encephalopathy (Ueda et al., 2015; Ding et al., 2022). The pathological features of CSF1R-microglial encephalopathy primarily include extensive white matter degeneration in the brain, diffuse loss of myelin, numerous neuroaxonal spheroids, and pigmented macrophages (Wong et al., 2011; Miura et al., 2018). *CSF1R* encodes a tyrosine kinase transmembrane receptor that regulates the proliferation, differentiation, and survival of mononuclear phagocytes, including microglia in the brain (Dulski et al., 2023). Upon stimulation by its ligands, colony-stimulating factor 1 (CSF1) or interleukin-34 (IL-34), the *CSF1R* protein forms homodimers and undergoes autophosphorylation at tyrosine residues in the kinase domain, activating downstream signaling pathways that lead to a cascade of related signal transduction events (Tsai et al., 2021). Research has shown that pathogenic mutations in *CSF1R* disrupt its autophosphorylation function, leading to impaired downstream signaling, which in turn affects the protein translation and transcription functions of microglia. This results in alterations in cell morphology and number, ultimately leading to the gradual degeneration of white matter in the brain. Therefore, *CSF1R* dysfunction is considered the primary cause of adult-onset leukodystrophy with neuroaxonal spheroids and pigmented glia (ALSP) (Rademakers et al., 2011). In the *CSF1R* mutation mouse model, dysfunction of microglial cells is manifested by abnormal intercellular communication, mitochondrial deformities, and enlarged lysosomes, which exacerbate brain inflammation and form a vicious cycle of immune-inflammation-induced aging. Furthermore, the *CSF1R* mutation not only affects the immune response of microglial cells but may also adversely affect the support and repair functions of neurons. The study found that in *CSF1R* mutant mice, the levels of inflammatory cytokines (such as IL-16 and IL-1β) were significantly elevated, indicating that microglial activation and pro-inflammatory responses exacerbate brain inflammation. This excessive immune response not only worsens the dysfunction of microglial cells but also correlates with the pathological processes of other neurodegenerative diseases, such as AD (Cheng et al., 2015; Wang et al., 2025).

Research has demonstrated that the association between *CSF1R* and AD primarily involves its regulation of microglial cell function and its modulation of neuroinflammatory responses. During the progression of AD, microglial cells respond to the accumulation of β-amyloid plaques and engage in their clearance. However, excessively activated microglial cells may induce an exaggerated inflammatory response, thereby exacerbating neuronal damage and accelerating disease progression. Research has demonstrated that certain mutations in the *CSF1R* gene, such as *p. P54Q, p. L536V, p. L868R, p. Q691H*, and *p. H703Y,* may increase the risk of AD. Furthermore, *CSF1R* has been identified as one of the molecular networks that regulate disease-associated microglial cells during the pathophysiology of AD (Hu et al., 2021; Xu et al., 2021). In this case, cerebrospinal fluid analysis showed a reduction in Aβ42, a decreased Aβ42/Aβ40 ratio, and an increase in t-tau protein, suggesting Alzheimer’s disease-related changes. A reduction in Aβ42 typically reflects a disruption in the β-amyloid clearance mechanism. Aβ42 is more prone to form amyloid plaques than Aβ40, and the decrease in the Aβ42/Aβ40 ratio further supports the hypothesis that microglial cells fail to effectively clear Aβ42, leading to the decline in the ratio. Meanwhile, the increase in t-tau protein suggests neuronal damage, which could be associated with the rapid progression of the disease. These changes in cerebrospinal fluid biomarkers may be linked to microglial dysfunction and the neurodegenerative progression induced by *CSF1R* mutations. Research by Hayer et al. has demonstrated that neurofilament light chain (NfL) is a reliable biomarker for CSF1R-associated leukoencephalopathy. NfL levels are markedly elevated in the cerebrospinal fluid of patients with CSF1R-microglial encephalopathy, exceeding the levels observed in the control group by more than 30 times (Hayer et al., 2018). Since NfL is a key component of the axonal cytoskeleton, the marked elevation of NfL levels indicates severe axonal damage in the patients (Khalil et al., 2024). However, in prior studies, cerebrospinal fluid levels of β-amyloid and phosphorylated tau protein were found to be normal in nearly all patients with CSF1R-associated microglial encephalopathy (Rosenstein et al., 2022), underscoring the distinctiveness of this case.

The imaging features of CSF1R-microglial encephalopathy encompass MRI-detected white matter lesions, predominantly found in the frontal-parietal lobes and periventricular regions, as well as cerebral atrophy (including corpus callosum thinning) (Mickeviciute et al., 2022), alongside persistent high signals on DWI. Over two-thirds of patients with CSF1R-microglial encephalopathy exhibit persistent high signals in white matter lesions on DWI (Ding et al., 2022). Existing studies suggest that persistent high signals on DWI represent the most distinguishing imaging feature of this disease. Unlike ischemic lesions, this feature can persist for months or even longer and may indicate neuronal demyelination or intra-myelin edema, which restricts the movement of water molecules, thereby leading to cytotoxic edema (Terasawa et al., 2013; Bender et al., 2014; Chen et al., 2020). Onder et al. proposed that the sustained presence of diffusion restriction in deep white matter lesions could serve as a key marker for CSF1R-microglial encephalopathy (Onder et al., 2020). Other studies also emphasize that persistent high DWI signals represent a critical radiological marker, strongly pointing to CSF1R-microglial encephalopathy and serving as an essential tool for differentiating this disease from other conditions (Li et al., 2020; Wu et al., 2022). In this case, however, the patient was diagnosed with CSF1R-microglial encephalopathy, but no notable high signals were detected on DWI. This further underscores the uniqueness of the case. The negative DWI in this case may reflect the clinical and imaging heterogeneity of CSF1R-microglial encephalopathy, implying that the imaging characteristics of this disease depend not only on acute changes in edema and demyelination, but may also be influenced by disease stage, genetic variations, and individual responses.

To date, 115 *CSF1R* mutation sites have been identified globally, including 93 missense mutations, 4 nonsense mutations, 4 insertions or deletions, 7 frameshift mutations, and 14 splice site mutations (Jiang et al., 2022). In this case, the patient carries a splice mutation (c.2654 + 1G>A) in the *CSF1R* gene, located in the intronic region of exon 20. This mutation occurs within the tyrosine kinase domain (TKD) and may disrupt the normal RNA splicing process, leading to the loss or alteration of *CSF1R* protein function (Leng et al., 2019). Notably, this mutation was previously identified in a Chinese family. Jiang et al. used bioinformatics tools such as Mutation Taster, FATHMM-XF, and CADD to predict that the c.2654 + 1G>A mutation is harmful and pathogenic. Additionally, dbscSNV classifies this splicing mutation as pathogenic, while predictions from Mutation Taster, NetGene2, and Human Splicing Finder suggest that it may disrupt the normal splicing process. According to the ACMG/AMP guidelines, the c.2654 + 1G>A mutation is potentially pathogenic (PVS1+PM2), but individuals carrying this mutation in the family have not exhibited the corresponding typical clinical manifestations (Jiang et al., 2022). Despite previous studies predicting the pathogenicity of the c.2654 + 1G>A mutation, there have been no reports of patients carrying this mutation who exhibit the corresponding clinical symptoms. This study presents, for the first time, a patient with this rare mutation and highlights the typical clinical manifestations, including early cognitive decline, personality changes, and behavioral abnormalities. In addition to these clinical features, our study reports an unusual imaging finding: unlike the typical high signal intensity on DWI observed in prior cases, our patient’s DWI was negative, suggesting variability in the radiological presentation of CSF1R-microglial encephalopathy. These findings not only provide direct clinical evidence of the mutation’s impact but also fill a critical gap in the existing research on both the clinical and imaging spectrum of this rare mutation. Although the patient’s son carries the same c.2654 + 1G>A mutation, he does not currently exhibit the same noticeable cognitive decline as the patient. However, the observed decline in delayed recall is still concerning. This could represent an early manifestation of CSF1R-microglial encephalopathy, particularly considering the mutation is inherited in an autosomal dominant manner. However, further longitudinal follow-up and neuropsychological testing are necessary to determine whether his cognitive function will decline in a manner similar to the patient’s. Given the unique genetic background, this study will emphasize long-term follow-up of the family.

In conclusion, when a patient presents with progressive cognitive decline at an early age of onset, a family history of the disease, and bilateral deep white matter lesions on brain MRI, despite the absence of significant high signals on DWI sequences, clinicians should remain vigilant for the possibility of hereditary white matter diseases, particularly CSF1R-microglial encephalopathy. *CSF1R* gene testing should be performed as early as possible to confirm the diagnosis and prevent misdiagnoses. Additionally, for genetically related diseases, testing should also be carried out for other family members to enable early diagnosis and intervention.

## Data Availability

The original contributions presented in the study are included in the article/supplementary material, further inquiries can be directed to the corresponding author.
